# Reprogramming of energy metabolism as a driver of aging

**DOI:** 10.18632/oncotarget.7645

**Published:** 2016-02-23

**Authors:** Zhaoyang Feng, Richard W. Hanson, Nathan A. Berger, Alexander Trubitsyn

**Affiliations:** ^1^ Department of Pharmacology, School of Medicine, Case Western Reserve University, Cleveland, OH, USA; ^2^ Department of Biochemistry, School of Medicine, Case Western Reserve University, Cleveland, OH, USA; ^3^ Case Comprehensive Cancer Center, School of Medicine, Case Western Reserve University, Cleveland, OH, USA; ^4^ Department of Medicine, School of Medicine, Case Western Reserve University, Cleveland, OH, USA; ^5^ Institute of Biology and Soil Sciences of Far Eastern Brach of Russian Academy of Science, Vladivostok, Russia

**Keywords:** aging, energy metabolism, mitochondria, glycolysis, physical activity, Gerotarget

## Abstract

Aging is characterized by progressive loss of cellular function and integrity. It has been thought to be driven by stochastic molecular damage. However, genetic and environmental maneuvers enhancing mitochondrial function or inhibiting glycolysis extend lifespan and promote healthy aging in many species. In post-fertile *Caenorhabditis elegans*, a progressive decline in phosphoenolpyruvate carboxykinase with age, and a reciprocal increase in pyruvate kinase shunt energy metabolism from oxidative metabolism to anaerobic glycolysis. This reduces the efficiency and total of energy generation. As a result, energy-dependent physical activity and other cellular functions decrease due to unmatched energy demand and supply. In return, decrease in physical activity accelerates this metabolic shift, forming a vicious cycle. This metabolic event is a determinant of aging, and is retarded by caloric restriction to counteract aging. In this review, we summarize these and other evidence supporting the idea that metabolic reprogramming is a driver of aging. We also suggest strategies to test this hypothesis

## INTRODUCTION

Aging is hallmarked by the progressive loss of cellular function and integrity that eventually leads to vulnerability and death of organisms [1]. It lowers the quality of life, and is a potent risk factor for cancer, diabetes, and other prevalent diseases [2]. It has been long thought that age-dependent accumulation of stochastic damage of molecules drives aging [3]. Current evidence demonstrate that a programmed event of energy metabolism is a determinant of aging that can be modified to modulate aging [4].

## AGING INVOLVES REPROGRAMMING OF ENERGY METABOLISM

Intermediary metabolism generates ATP from nutrients, providing energy for cellular function and maintenance. Alterations in energy metabolism are linked to the aging process and aging-associated diseases [5]. Substantial evidence has demonstrated that energy production progressively decreases with age in all organisms, mainly due to the decline in the function of mitochondria [6]. Aged organisms also exhibit disrupted homeostasis of carbohydrates, amino acids, and fatty acids [5], major biological fuels [7]. The exact alterations in energy metabolism that are associated with aging, their physiological impact, and their contribution to aging are unclear, impeding the understanding of aging mechanisms and the development of mechanism-based strategies to modulate aging.

The decline in mitochondrial function with age has been attributed to the accumulation of stochastic damage to mitochondrial DNA [3], primarily by reactive oxygen species produced through the electron transport chain (ETC) during ATP production. Although oxidative damage of mitochondrial DNA accumulates with age [8, 9] and leads to reduced gene expression [10, 11], it is inconclusive whether oxidative damage is the cause of aging-associated decline in mitochondrial function or aging [12, 13]. Recently, we have reported that the aging of *C. elegans*, a genetic model of aging that lives about three weeks, is highlighted by a progressive decline in cytosolic phosphoenolpyruvate carboxykinase (PEPCK-C) after the reproductive peak, and a reciprocal increase in pyruvate kinase (PK) [4]. While PK is an enzyme of glycolysis, PEPCK-C is a metabolic enzyme associated with longevity [4, 14, 15]. A key consequence of this metabolic event is the shunt of energy metabolism from oxidative metabolism to anaerobic glycolysis. In all aerobic species, ATP can be generated both in the presence and absence of oxygen. But 30-36 ATP can be generated from one glucose molecule through oxidative metabolism, while only 2 ATP are produced by anaerobic glycolysis [7]. As a result, reciprocal changes in PEPCK-C and PK with age reduce the efficiency of and total energy production [4].

**Figure 1:**
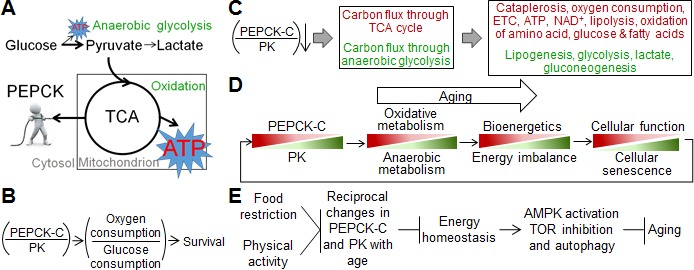
Models for aging-associated changes in energy metabolism and aging **A**., “PEPCK-C pulls the strings”, schema illustrating a role of PEPCK-C in energy metabolism. **B**.-**C**., Schema illustrating the impact of the ratio of PEPCK-C over PK on energy metabolism and survival. Red, decrease; green increase. **D**.-**E**., Models for the role of reciprocal changes in PEPCK-C and PK in aging. These metabolic changes may also promote aging via reduced carbon supply from the TCA cycle, which is needed for various biosynthetic pathways. Direct evidence supporting this view, however, is currently lacking. Panels D-E were originally published in Journal of Biological Chemistry, Yuan, *et al*., Reciprocal changes in phosphoenolpyruvate carboxykinase and pyruvate kinase with age are a determinant of aging in *Caenorhabditis elegans*. J Biol Chem, 291: 1307-19. © the American Society for Biochemistry and Molecular Biology.

How do changes in PEPCK-C and PK, two cytosolic enzymes, lead to decline in mitochondrial function? The core of energy metabolism is the tricarboxylic acid (TCA) cycle [7], a series of chemical reactions that oxidize carbohydrates, fats and proteins into carbon dioxide, generating NADH that is used by ETC to produce ATP. Moreover, the TCA cycle intermediates can be withdrawn from mitochondria to the cytosol, a process called cataplerosis. This supplies carbons for the synthesis of glyceride-glycerol, serine, and glucose, as well as metabolites derived from these chemicals [16]. Traditionally viewed as a rate-limiting enzyme of gluconeogenesis and glyceroneogenesis, PEPCK-C is a major cataplerotic enzyme that links the TCA cycle with the metabolism of carbohydrates, fatty acids, amino acids, and other metabolites [16]. At the biochemical level, the activity of PEPCK-C is correlated tightly with the flux of the TCA cycle but not gluconeogenesis [17]. At the organismal level, PEPCK-C is required for the integration of energy metabolism [18], and is critical for the homeostasis of glucose, fatty acids, amino acids and other related metabolites (see ref [16] for review). In *C. elegans*, PEPCK-C accelerates TCA cycle flux and promotes oxidative metabolism [4], likely via increased cataplerosis (Figure 1A). PEPCK-C also increases mitochondrial respiration and counteracts its decline with age. On the other hand, PK is a key glycolytic enzyme that greatly favors the conversion of phosphoenolpyruvate to pyruvate, promoting glycolysis [7]. During energy production, pyruvate either enters mitochondria for oxidation, or is converted to lactate by lactate dehydrogenase. PEPCK-C shunts glucose metabolism toward oxidation, reducing lactate production [4].

The reciprocal changes in PEPCK-C and PK with age, and their impact on oxidative metabolism and anaerobic glycolysis are likely conserved from *C. elegans* to humans. First, skeletal muscle of aging humans from 10s to 70s exhibits an aging-associated decrease in PEPCK-C activity, and increases in PK and lactate dehydrogenase that cannot be explained by alterations in numerical ratio of type I and type II muscle fibers with age [19]. In aged skeletal muscle and liver of mammalian animals, *PEPCK-C* mRNA is decreased and *PK* mRNA is increased [20, 21]. Second, aging mammals including humans display decreased mitochondrial function and increased glycolysis in many tissues such as liver, skeletal muscle and brain [22-25], as well as elevated lactate in both tissues and serum [26]. Moreover, platelets of aged humans exhibit reduced ATP production by mitochondria, and increased ATP production by anaerobic glycolysis [27], which likely reflects aging-associated changes in energy metabolism of the whole body [27, 28].

## RECIPROCAL CHANGES IN PEPCK-C AND PK PROFOUNDLY IMPACT AGING ORGANISMS

What are the physiological effects of reciprocal changes of PEPCK-C and PK with age? The decline in mitochondrial bioenergetics may subject aging organisms to a relative energy deficiency, although the PK-driven increase in glycolysis likely compensates for some of the reduced energy production. A deficit in energy supply reduces the function and integrity of many cells and tissues, hence the survival of organisms, due to unmatched energy demand and supply (Figure 1B). In support of this view, PEPCK-C promotes physical activity, fertility, autophagy, defense against osmotic and oxidative stresses, and many other energy consuming processes in various animal species [4, 14, 15, 29-34]. During the aging of *C. elegans*, decline in PEPCK-C is coupled with loss of physical activity, a major energy consumer [35], and genetically enhanced PEPCK-C preserves physical activity and extends lifespan in a dose-dependent manner [4]. Of note, PEPCK-C promotes physical activity to increase ATP turnover, AMP/ATP ratio (a key indicator of cellular energy demand), the activation of 5′ AMP kinase (AMPK, a major mediator of energy homeostasis activated by higher AMP:ATP ratio [36]), fuel oxidation, ATP content, and food intake, both acutely and chronically. Many of these effects of PEPCK-C on energy demand and supply, and cellular function and maintenance require the activation of AMPK [4].

In addition to decline in energy production, the following alterations in energy metabolism are predicted, based on the metabolic roles of PEPCK-C, PK, and the TCA cycle: 1) increase in synthesis and deposition of fats; 2) disrupted homeostasis of glucose and amino acids; 3) reduction in NAD^+^; and 4) reduction in biosynthesis associated with cataplerosis (Figure 1C).

Unlike glucose, fats can only be oxidized to produce ATP [7]. The reduction in oxidative metabolism with age would lead to decreased utilization of fats. On the other hand, increased PK activity and glycolytic flux in aged organisms should produce more pyruvate. In addition to being oxidized in mitochondria or converted to lactate, pyruvate can serve as a precursor of lipogenesis or gluconeogenesis. Because mitochondrial function is reduced, more pyruvate would be shunt to lipogenesis or gluconeogenesis. Indeed, aging involves a shift of fatty metabolism toward lipogenesis in mice [37] and accumulation of fats in all organisms including humans [38]. In mice, decline in PEPCK-C with age underlies aging-associated reduction in lipolysis and the coordinative down regulation of mitochondrial enzymes [39]. Indeed, aged mice over-expressing PEPCK-C exhibited less subcutaneous, visceral, and pericardial fat deposit than even younger control mice fed the same regular chow diet [31]

The shift of energy metabolism from oxidative metabolism to anaerobic glycolysis suggests that aged organisms demand more glucose as energy source. Consistent with this view, gluconeogenesis is elevated in aged yeast [40]. In aged mammals, basal gluconeogenic capacity and blood glucose produced through gluconeogenesis are increased [41], while hepatic incorporation of glucose to glycogen is decreased [42]. In contrast to gluconeogenesis, glucose uptake into skeletal muscle, brain and other energy consuming tissues decreases with age due to reduced insulin signaling [43], decreased insulin sensitivity [44], and reduced glucose transporters [45]. An increased demand and supply of glucose, while a reduction in usable glucose may contribute to the disrupted homeostasis of glucose in aged organisms [46]. Sarcopenia is the progressive loss of muscle in aging humans and animals [47]. The lost muscle mass, primarily proteins, results in increased alanine and glutamine in circulation [48]. A fate of these amino acids is to be oxidized in mitochondria. The shift of energy metabolism away from oxidative metabolism would reduce the disposal of these amino acids, and contribute to the disrupted homeostasis of amino acids in aged organisms [49].

Redox homeostasis is critical for cellular function and integrity [50]. Significantly, aging is accompanied by a progressive decline in intracellular NAD^+^ in species including humans [51, 52]. NAD^+^ is an essential cofactor for sirtuin, a deacetylase that promotes longevity and healthy aging [53]. It is also a necessary substrate for poly (ADP-ribose) polymerase [54], a critical enzyme in the DNA repair process [54-56]. Decreased NAD^+^ increases the vulnerability of cells to accumulation of DNA damage with age [57-59]. The breakdown of glucose to pyruvate consumes NAD^+^ [7]. NAD^+^ is regenerated through fermentation that converts pyruvate to lactate. Increased glycolytic flux in aged organisms would lead to increased consumption of NAD^+^. Insufficient regeneration of NAD^+^ could result in reduction in NAD^+^.

The cataplerotic role of PEPCK-C has been recognized in cell proliferation [60], and adaptive response to stresses, such as acidosis [61], inflammation [62], and osmotic stress [30]. Under these circumstances, PEPCK-C supports the synthesis of ribose, glucose, steroid, and glycerol from amino acids and other non-carbohydrates. The released ammonia from the oxidation of amino acids relieves acidosis. Likely, decline in PEPCK-C with age reduces cataplerosis and its related biosynthesis, contributing to reduced proliferation and increased vulnerability associated with aging.

Most cancer cells exhibit the Warburg effect [63], higher glycolysis followed by lactate production, instead of lower glycolysis followed by oxidation. Type II diabetes is associated with metabolic changes paralleling the Warburg effect, including expression of genes involved in anaerobic glycolysis [64] and a decline in mitochondrial function [65]. Thus, the shift of energy metabolism from oxidative metabolism to glycolysis driven by reciprocal changes in PEPCK-C and PK with age may promote tumorigenesis and the prevalence of diabetes [66-68]. For example, metformin, the most commonly prescribed drug treating type II diabetes, may shunt energy metabolism toward oxidative metabolism and away from glycolysis to counteract cancers [68].

Collectively, the cataplerotic role of PEPCK-C acts as a major adaptor of energy metabolism, and a carbon valve for various biosynthetic pathways. Reciprocal changes in PEPCK-C and PK after the reproductive peak are a lead metabolic event associated with aging. This event re-patterns metabolism of aging organisms, decreases their cellular function and integrity, and promotes the onset of aging-associated diseases.

## RECIPROCAL CHANGES IN PEPCK-C AND PK WITH AGE DETERMINE AGING

Evidence from mice and *C. elegans* demonstrate that reciprocal changes in PEPCK-C and PK with age determine aging. First, PEPCK-C counteracts loss of cellular function and integrity with age. Specifically, it extends fertility, retards aging-associated decrease in physical activity, a negative indicator of health span and lifespan [69], and enhances autophagic activity [4, 14]. Autophagy is a cell repair mechanism that removes molecular wastes, and counteracts aging and aging-related diseases [70-73]. Second, PEPCK-C retards cellular senescence [4], assessed by the accumulation of molecular wastes such as lipofuscin and β-galactosidase [74, 75], and the expression of the proliferation restrictive marker cyclin kinase inhibitor [76]. Cellular senescence may contribute to aging [77]. Moreover, reciprocal changes in PEPCK-C and PK with age are necessary and sufficient to limit lifespan and fertility [4]. Last, PEPCK-C activity is correlated with lifespan, and its enzyme level predicts life expectancy [4].

Many effects of PEPCK-C on aging including lifespan extension require the activation of AMPK signaling and/or the inhibition of Target of Rapamycin (TOR) signaling [4]. AMPK and TOR signaling are two major molecular signals that control aging in species including mammals [78-80]. The beneficial impact of activation of AMPK and inhibition of TOR on lifespan necessitates autophagy [81-83]. Consistently, PEPCK-C enhances the activity of autophagy in aged *C. elegans*, and requires the expression of autophagic genes to promote longevity [4]. Interestingly, AMPK primarily drives catabolism that produces energy and promotes mitochondrial oxidation, and is a negative regulator of glycolysis [84]. On the other hand, TOR signaling is a cellular energy sensor of excessive nutrient and energy that upregulates PK and glycolysis [85]. The anti-aging effects mediated by TOR inhibition involve increased energy metabolism and oxidative phosphorylation complex [86-90].

In summary, reciprocal changes in PEPCK-C and PK with age are a determinant of aging. The mechanisms include disrupted energy homeostasis, as well as altered AMPK, TOR signaling, and autophagy. Consistently, mitochondrial bioenergetics and autophagic activity are preserved in fibroblasts of centenarians [91]. Intriguingly, declines in PEPCK-C and physical activity with age promote each other to limit reproductive life and lifespan [4] via a feedback mechanism [4, 14, 92, 93]. These observations indicate that a vicious cycle of reciprocal changes in PEPCK-C and PK, and decline in cellular function and integrity drives aging (Figure 1D). The causality between changes in metabolism and in cellular function and integrity is unknown, and is likely a “chicken and egg” dilemma.

## RECIPROCAL CHANGES IN PEPCK-C AND PK ARE A COMMON DENOMINATOR OF AGING

Molecular signals, pharmacological reagents, appropriate environmental stresses, and calorie restriction (CR) extend lifespan and may also improve health in various species. Strikingly, most of these maneuvers either increase oxidative metabolism and energy production, inhibit glycolysis, or both. In addition to AMPK and TOR, insulin/IGF signaling (IIS) [94] and sirtuin [53] are molecular signals that affect lifespan. Reduced IIS [95-99] and sirtuin [51, 100-103] increase or stabilize PEPCK-C, promote oxidative metabolism, and inhibit PK and/or glycolysis.

Metformin is an indirect activator of AMPK, and rapamycin is an inhibitor of TOR signaling. Metformin [104, 105], rapamycin [106, 107] and inhibitors of glycolysis, such as D-glucosamine and 2-Deoxy-D-glucose [108, 109], extend lifespan in many species including mammals. D-glucosamine and 2-Deoxy-D-glucose increase mitochondrial respiration, and require AMPK to extend lifespan [108, 109], suggesting that a shunt of energy metabolism toward oxidative metabolism is critical for the observed lifespan extension. Besides pharmacological reagents, lower ambient temperature, and osmotic and oxidative stresses increase PEPCK-C and/or oxidative metabolism, and extend lifespan in *C. elegans* and other lower organisms [16, 30, 110-112].

CR is the most robust intervention that extends lifespan and improves health in species ranging from yeast to non-human primates, via AMPK-TOR-autophagy axis [80-83]. CR increases PEPCK-C activity and oxidative metabolism while inhibiting PK activity and glycolysis in animals [15, 113-116]. In humans, CR increases mitochondrial biogenesis [117]. A plausible biological reason underling this metabolic shift is to promote efficient energy production and cataplerosis, in order to meet the energy [118] and biosynthetic [119-121] need under limited resources. Significantly, CR counteracts reciprocal changes in PEPCK-C and PK with age to elicit anti-aging effects including longevity in *C. elegans* [4]. On the other hand, physical activity, which extends life expectancy in humans [122], increases energy expenditure, PEPCK-C and mitochondrial function. Notably, both mice and *C. elegans* over-expressing PEPCK-C exhibited increased physical activity, ate more, weighed less, had extended fertility, and lived longer [4, 14]. Thus, energy balance, achieved by reduced “energy in” from CR, enhanced “energy out” from enhanced physical activity, or their combination, counteracts reciprocal changes in PEPCK-C and PK with age to retard aging.

In summary, reciprocal changes in PEPCK-C and PK activity with age, and the consequent shift of energy metabolism are a common denominator of aging. These alterations can be retarded by CR, CR mimetics, and other genetic and environmental factors to counteract aging via AMPK and TOR pathways (Figure 1E).

## CONCLUSIONS AND FUTURE PERSPECTIVE

Reciprocal changes in PEPCK-C and PK with age are likely part of a bigger reprogramming of energy metabolism that profoundly affects the physiology of aging organisms, thereby impacting the aging process. It is important to obtain a complete picture of changes in metabolism with age, and their influence on decline in cellular function, cellular senescence, lifespan, and other aging traits. Such investigation should focus on metabolic pathways moving carbons into and out of the TCA cycle, and those affecting the homeostasis of glucose, fats and amino acids. This is because both CR and an optimized ratio of macronutrients (carbohydrates, proteins and fats) without reduction in total calorie intake extend lifespan in mice [123-125], suggesting that alterations in these metabolic pathways have significant but complex impact on aging.

Evidence outlined here support the bioenergetics theory of aging [126, 127], which proposes that the decline in bioenergetics with age is the driver of aging. Specifically, the decline in bioenergetics with age is a pacemaker in the aging process, whereas other aging-associated phenomena, such as the accumulation of reactive species and the decline in repair mechanisms, are secondary to the decline in bioenergetics. The sum of all these changes leads to loss of physiological function and eventually vulnerability and death of organisms. The bioenergetics theory of aging also hypothesizes that an unidentified primary genetic program (aging clock) controls the decline in bioenergetics. However, the presence of a vicious cycle between reciprocal changes in PEPCK-C and PK, and decline in cellular function and integrity indicates that a genetically programmed aging clock may [128] or may not [129-131] be needed for the decline in bioenergetics with age. For example, a quasi-programmed hyperfunction, which has been proposed to be associated with development and have harmful impact on organisms, may start this vicious cycle without a genetically programmed aging clock; while the pace of this vicious cycle may be mediated by a genetic program. In either case, it is critical to understand the mechanisms underlying aging-associated changes in PEPCK-C and PK, which are currently unknown. Remarkably, PEPCK-C is acetylated in yeast [132] and human cells [133], and PEPCK-C acetylation leads to its degradation [100]. Sirtuin, which promotes longevity in many species, deacetylates and stabilizes PEPCK-C [100]. Deacetylation mediated by sirtuin shunts energy metabolism away from glycolysis and toward oxidative metabolism [134-137], promoting energy production [138]. Sirtuin expression and activity decrease with age [139, 140], and CR [141] and exercise [142] increase sirtuin to decrease protein acetylation. It will be interesting to examine if PEPCK-C acetylation is involved in its decline with age, if sirtuin slows this decline, and if sirtuin retards aging via altered PEPCK-C stability.

Last, enhancing PEPCK-C is sufficient to delay many key aging-associated metabolic and physiological changes and increase lifespan [4, 14, 15]. Thus interventions that sustain PEPCK-C represent a novel strategy to counteract aging.
